# Invasive lobular carcinoma of the breast: metastatic patterns and treatment modalities—a review

**DOI:** 10.3389/fonc.2025.1631670

**Published:** 2025-09-26

**Authors:** Bixin Yu, Li Yan, HongYan Wang, Jin Yang, Jiao Yang

**Affiliations:** ^1^ Department of Medical Oncology, The First Affiliated Hospital of Xi’an Jiaotong University, Xi’an, Shaanxi, China; ^2^ Department of Pathology, The First Affiliated Hospital of Xi’an Jiao-tong University, Xi’an, Shaanxi, China; ^3^ Cancer Center, The First Affiliated Hospital of Xi’an Jiaotong Univer-sity, Xi’an, Shaanxi, China; ^4^ Phase I Clinical Trial Research Center, The First Affiliated Hospital of Xi’an Jiaotong University, Xi’an, Shaanxi, China; ^5^ Precision Medicine Center, The First Affiliated Hospital of Xi’an Jiao-tong University, Xi’an, Shaanxi, China

**Keywords:** invasive lobular carcinoma, metastatic pattern, prognosis, treatment mode, endocrine resistance

## Abstract

Compared with invasive ductal carcinoma (IDC), invasive lobular carcinoma (ILC) exhibits distinct histologic, molecular, and clinical characteristics, including unique metastatic patterns. This review focuses on three major aspects: (1) an analysis of metastatic behavior across different ILC histologic subtypes, with a preliminary exploration of potential correlations with molecular features; (2) a synthesis of current treatment strategies, highlighting challenges such as endocrine resistance, limited tailored protocols, and emerging immunotherapeutic opportunities; and (3) a review of clinical trials from 2022 to 2024 to identify evolving strategies and future directions for individualized therapy. By integrating pathology, molecular profiling, and clinical data, this review emphasizes ILC’s distinctive metastatic behavior and unmet clinical needs, providing a conceptual framework to guide future translational research and therapeutic development.

## 1 Introduction

Invasive lobular carcinoma(ILC) is the second most common breast cancer histologic subtype, accounting for up to 15% of all cases, following invasive ductal carcinoma(IDC) (1). In recent years, the apparent increase in incidence of invasive lobular carcinoma (ILC) may reflect not only hormonal risk factors (e.g., menopausal hormone-replacement therapy) but also advances in diagnostic imaging and screening protocols (2–4). In terms of clinical presentation, patients with ILC tend to have a larger mass size at the time of diagnosis, older age of onset, lower histologic grade, later clinical stage, a higher incidence of bilateral and multicentricity lesions compared to patients with IDC (5–7).

Some studies on bilateral primary breast cancer suggest a high probability of lobular histology and hormone receptor(HR) positivity (8, 9). ILC tends to invade the mesenchyme as a monolayer of cells, which tend to be very dispersed, thus presenting as multicentric and difficult to detect on palpation and imaging (10). ILC exhibits a higher propensity for lymph node (LN)invasion (11), in which some LN metastases present as occult metastasis, indicating the difficulty in diagnosis and staging (12). However, the relationship between such metastasis and prognosis is still unclear (13). In terms of distant metastatic pattern, ILCs spread predominantly to the bones and liver, while IDCs metastasize more frequently to the lungs and liver (14). Most ILCs are treated based on their hormone receptor status, similar to IDCs. However, due to the biological differences between the two subtypes, tailored treatment strategies for ILC are still needed.

## 2 Specific metastatic pattern of invasive lobular carcinoma

### 2.1 Unique metastatic characteristics and mechanisms

The metastatic pattern of ILC is unusual compared to that of IDC, which tends to be more extensive and tends to invade the plasma membrane layer (1). While bone and liver are common metastatic sites for both subtypes, ILC more frequently involves the gastrointestinal tract, peritoneum, retroperitoneum, and genitourinary system — with the ovary being a particularly recognized site — than IDC according to several retrospective analyses (15–17). ILCs also invade the central nervous system, such as the meninges, where carcinomatous meningitis occurs almost exclusively in patients with ILC (18, 19). Metastases to rare sites such as the orbit (20, 21), parotid gland (22), perianal area (23), and even intratumoral also happen (24, 25). The hallmark loss of E-cadherin (CDH1 mutation) impairs epithelial cohesion, contributing to the characteristic discohesive infiltration pattern of ILC (26, 27). This alteration can activate downstream pathways such as Rho/ROCK, which may facilitate tumor cell survival and dissemination (28). Nevertheless, these mechanisms are insufficient to fully account for its predilection for metastasis to specific sites, indicating that additional molecular and microenvironmental factors are likely involved and require further elucidation.

### 2.2 Histologic and molecular subtypes and their association with metastatic patterns in ILC

Invasive lobular carcinoma (ILC) encompasses a spectrum of histologic subtypes with distinct biological behavior, molecular alterations, and metastatic patterns. According to the WHO Classification of Tumours of the Breast (29), formally recognized morphologic variants include classic, mixed, alveolar, solid, tubulolobular, and pleomorphic types. In addition to these WHO-recognized subtypes, we also included trabecular, histiocytoid, and apocrine (apocrine/sweat gland-like) morphologies, as well as signet-ring cell differentiation, which are described in the literature but not listed as formal WHO diagnostic categories, as part of a hypothesis-driven risk stratification model in this review (30–35).

**Figure 1:**
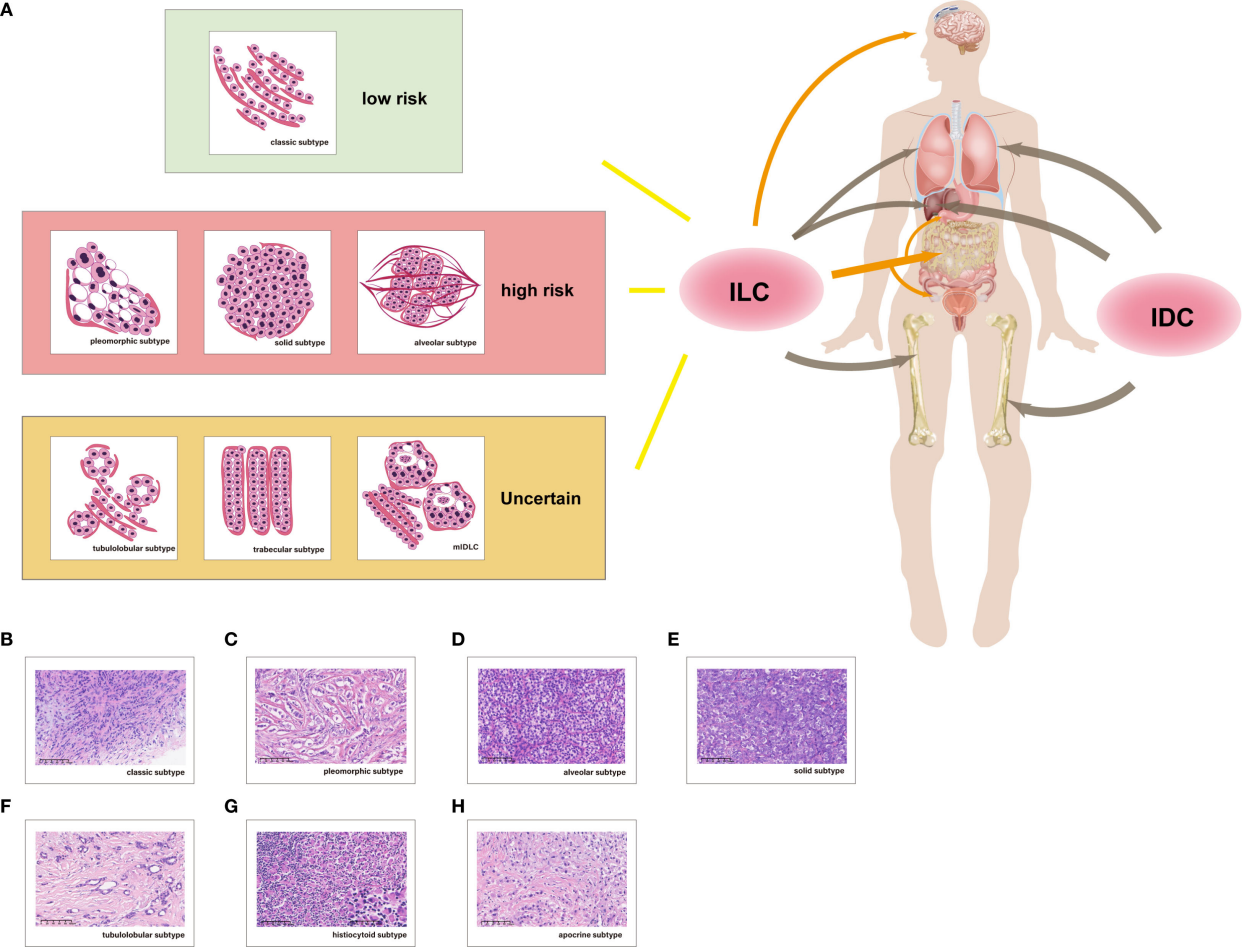
Risk-based histologic stratification of ILC and its broader metastatic profile compared to IDC. **(A)** Risk stratification and metastatic features of ILC: Classic ILC is classified as low-risk, whereas alveolar, pleomorphic, and solid ILC are considered high-risk due to their more aggressive histologic features. Tubulolobular, mixed (mIDLC), and trabecular ILC are assigned to an uncertain-risk category, reflecting limited or heterogeneous evidence. Apocrine and histiocytoid variants are extremely rare and not included in the risk stratification. Unlike IDC, which predominantly metastasizes to bone, liver, and lung, ILC exhibits a broader spectrum of metastatic dissemination, frequently involving the peritoneum, retroperitoneum, gastrointestinal tract, and genitourinary system. These distinctive patterns warrant increased clinical vigilance and potentially adapted surveillance strategies for ILC patients. **(B–H)** Representative histology of ILC variants: **(B)** Classic – small, uniform cells in single-file cords; **(C)** Pleomorphic – high-grade cells with nuclear atypia and prominent nucleoli; **(D)** Alveolar – nests of tumor cells separated by thin septa; **(E)** Solid – broad sheets of discohesive cells; **(F)** Tubulolobular – combination of small tubules and single-file growth; **(G)** Histiocytoid – abundant foamy cytoplasm, bland nuclei; **(H)** Apocrine – large polygonal cells with granular eosinophilic cytoplasm and prominent nucleoli; Signet-ring differentiation is indicated as a morphologic feature rather than a distinct subtype; mixed/mIDLC shows pronounced heterogeneity, and trabecular ILC is exceedingly rare.

Classic ILC (cILC) is the most prevalent subtype and typically displays small, uniform tumor cells with round nuclei, arranged in single-file linear cords or targetoid periductal patterns, often with absent or minimal desmoplastic response (29, 31) (**Figure 1B**). These tumors are frequently associated with loss of E-cadherin expression and show luminal A molecular features, including strong estrogen receptor (ER) positivity and low proliferative index (31, 36). As the dominant subtype, cILC likely contributes to the bone-predominant metastatic pattern observed in ILC overall (15, 31).

Pleomorphic lobular carcinoma (PLC), by contrast, is a high-grade variant characterized by marked nuclear pleomorphism, increased nuclear size and irregularity, and frequent mitotic figures (29) (**Figure 1C**). Cells may retain the discohesive growth pattern typical of lobular carcinoma but exhibit prominent nucleoli and abundant eosinophilic cytoplasm (37). Compared to cILC and IDC, PLC exhibits a higher proportion of HER2-positive and triple-negative subtypes, while maintaining a considerable proportion of ER-positive tumors (38, 39). Molecularly, PLCs are enriched for p53 mutations, and show higher rates of HER2 overexpression and PIK3CA alterations compared to cILC (39–41). These features correlate with the aggressive clinical behavior of PLC, which shows a higher propensity for visceral and brain metastases in HER2-positive cases, whereas HR-positive and triple-negative subtypes more commonly metastasize to bone (38, 42), making it a high-risk subtype. Alveolar ILC is composed of small tumor cells arranged in nests of more than 20 cells, separated by thin fibrous septa (29) (**Figure 1D**). This pattern may mimic lobular hyperplasia, posing diagnostic challenges (43). Despite earlier large cohort studies suggesting outcomes closer to cILC (44), the compact cell nests and more aggressive histologic appearance of alveolar ILC support it as representing a higher-risk phenotype (**Figure 1D**). In the solid ILC, tumor cells infiltrate the surrounding tissue in broad solid sheets, with minimal intervening stroma (29). This variant has been associated with more aggressive features and worse prognosis (45), and is therefore considered high-risk. However, there are insufficient data to clearly link alveolar or solid ILC to specific metastatic sites.

Tubulolobular carcinoma (TLC), currently classified by the WHO under the spectrum of ILC, exhibits both small tubular structures and single-file lobular growth, representing an intermediate morphology between ductal and lobular carcinoma (29) (**Figure 1F**). However, its nosological position remains debated, with some considering TLC a variant of ILC and others regarding it as closer to IDC (46–48). Its metastatic profile is poorly characterized, though limited reports suggest possible associations with gastrointestinal involvement (49, 50). Mixed invasive ductal-lobular carcinoma (mIDLC), defined by WHO as tumors with 10–90% lobular components admixed with at least 10% ductal features (29), exhibits heterogeneous morphology and variable clinical outcomes. It has been suggested that mIDLC is more similar to ILC histologically, but has a smaller tumor size and better sensitivity to neoadjuvant chemotherapy than ILC (51). However, some studies have shown that mIDLC is often detected with a larger mass, more advanced stage, higher lymph node grade, and higher rate of bone metastasis, with little difference in outcome of ILC and IDC (36). Bone metastasis is common, while peritoneal involvement appears less frequent than in pure ILC (52, 53). Due to the relatively low incidence of mIDLC, existing studies are often constrained by small sample sizes and inconsistent findings. Further research with larger, stratified cohorts is warranted to better define its biological behavior and clinical significance.

Rare ILC variants not formally recognized by WHO include trabecular, histiocytoid, apocrine and signet-ring cell types. Trabecular ILC consists of cells arranged in cord-like or trabecular patterns (45). Histiocytoid ILC is characterized by abundant foamy cytoplasm and relatively bland nuclei, which may occasionally mimic benign histiocytic lesions (**Figure 1G**), and has been reported to metastasize to orbital or cutaneous sites (54). Apocrine differentiation in invasive lobular carcinoma (ILC) is rare and characterized by large polygonal cells with granular eosinophilic cytoplasm, prominent nucleoli, and distinct cell borders (30, 55) (**Figure 1H**). These tumors frequently express androgen receptor (AR) (56). Most of the available morphologic and molecular features are extrapolated from studies of apocrine breast carcinoma in general, which predominantly comprises ductal histology, and thus the clinical significance of apocrine ILC remains extremely limited. Signet-ring cell differentiation is characterized by intracytoplasmic mucin displacing the nucleus and has been reported to metastasize to gastrointestinal and gynecologic organs (57–59), which is considered a differentiation pattern rather than a distinct subtype (60). Collectively, these rare variants illustrate the morphological heterogeneity of ILC beyond WHO-recognized subtypes, though evidence is limited and robust conclusions about prognostic or metastatic patterns are difficult to draw.

Based on the above, we propose a simplified tentative framework.

- Low-risk: Classic ILC, characterized by low-grade morphology, strong ER expression, bone-predominant metastases, and favorable prognosis.
- High-risk: Pleomorphic and solid variants, and tentatively alveolar ILC, reflecting their aggressive histology, higher grade, and increased risk of visceral and CNS metastases.
- Uncertain risk: Tubulolobular, mixed, and trabecular subtypes, due to nosological controversy, morphologic heterogeneity, and limited prognostic data.

This provisional framework aims to prompt future validation in large, subtype-stratified cohorts. In clinical practice, recognizing aggressive variants may support more vigilant imaging, earlier CNS screening, or extended surveillance, especially for high-risk subtypes. Conversely, low-risk subtypes might allow de-escalated strategies in selected patients. While evidence linking specific histologic subtypes to defined metastatic sites remains limited, current data support biological heterogeneity. We thus propose a conceptual stratification by recurrence risk and highlight the broader metastatic spectrum of ILC compared to IDC (**Figure 1**).

Molecularly, most ILCs are HER2-negative and enriched in luminal A subtype (61). Triple-negative ILCs (TN-ILC), though rare (2–9% of ILCs), tend to present at advanced stage and have poor long-term survival (1, 62). Interestingly, TN−ILCs often express AR at higher levels than TN−IDC, displaying an AR−driven transcriptomic profile (63). While this has drawn therapeutic interest, clinical data remain very limited, and the potential benefit is still uncertain. Moreover, the well-recognized proclivity of ILC for bone metastasis may likewise reflect its predominance within hormone receptor-positive (HR+) breast cancer. Clinical and molecular studies consistently show that HR+ (ER-positive) tumors are significantly more likely to colonize bone compared to HR-negative subtypes, suggesting a biological preference driven by hormone receptor biology (64, 65). Experimental insights further indicate that ER signaling contributes to bone homeostasis and may create a niche favorable to metastatic growth (65). Currently, due to the rarity of HER2-positive and triple-negative ILC, there is a lack of sufficient studies specifically investigating the impact of these molecular subtypes on metastatic sites in ILC. Inferences regarding the potential influence of HER2-positive or triple-negative status on ILC metastasis are largely extrapolated from observations in corresponding ductal carcinoma subtypes.

Taken together, the metastatic behavior of ILC appears to be shaped by both histologic and molecular subtypes. At present, the interplay between histologic subtype, molecular subtype, and metastatic organotropism in ILC remains incompletely understood. It is therefore more reasonable to consider that the metastatic and prognostic features of ILC are collectively driven by multiple factors, including tumor grade, histologic classification, molecular subtype, and TNM stage. This highlights the need for larger, well-characterized cohorts to disentangle the relative contribution of each dimension.

## 3 Clinical management and molecular therapeutic opportunities in ILC

### 3.1 Management of early-stage ILC

#### 3.1.1 Neoadjuvant therapy

##### 3.1.1.1 Neoadjuvant chemotherapy

Compared to IDC, ILC is significantly less sensitive to NACT and derives limited clinical benefit (66). Multiple studies report low pathological complete response (pCR) rates in both primary tumors and axillary nodes following NACT in ILC patients (67–69). Moreover, NACT does not significantly improve downstaging rates, and a substantial proportion of patients still require mastectomy despite preoperative chemotherapy (70, 71). This is likely attributed to the predominance of the luminal A subtype in ILC, which is hormone receptor (HR)-positive and HER2-negative (72, 73). Subgroups more likely to benefit from NACT include tumors with clinical stage T2/T3, HR-negative, ER-positive but PR-negative status, histologic high-grade features, or atypical lobular morphology (69, 74, 75). Quirke et al. found that patients with atypical ILC (e.g., HR-/HER2+ or pleomorphic ILC) responded better than classic ILC (HR+/HER2-) to NACT (76). Möbus et al. (GAIN-2 trial) and Schneeweiss et al. (GeparOcto trial) both explored the role of dose-dense chemotherapy in HR+/HER2− breast cancer. Subgroup analyses suggested that a subset of patients—particularly those under 50 years of age and with invasive lobular carcinoma—may derive benefit from intensified regimens such as iddEPC, although the study populations and benefit profiles were not fully overlapping (77, 78). HR-positive/HER2-negative patients account for the majority of patients with ILC, which may imply that dose-intensive chemotherapy can be attempted in patients with ILC, but the age of the patient and tolerability still need to be considered. While dose-dense chemotherapy may be effective for selected ILC subtypes, current evidence remains insufficient to support broad application of NACT, highlighting the need for subtype-specific trials.

##### 3.1.1.2 Neoadjuvant endocrine therapy

In recent years, the use of NET has been on the agenda due to the high expression of hormone receptors in ILC (79). Several large clinical studies have shown that NET can be effective in tumor control and stage reduction in hormone receptor-positive, Her2-negative invasive breast cancer, both before and after menopause (80–82). According to U.S. Cancer Database analysis, use of NET in clinical T2 ILC increased from 2.1% in 2010 to 5% in 2016, suggesting growing acceptance (83). Longer NET durations are associated with increased breast conservation rates and fewer axillary dissections (79, 83–85). Adding CDK4/6 inhibitors (e.g., Palbociclib) to NET enhances proliferation suppression and may allow patients with luminal subtypes to avoid chemotherapy toxicity (86, 87). In NEOPAL trial, researchers found that neoadjuvant letrozole-Palbociclib strategy may allow some patients with luminal breast cancer to be able to spare chemotherapy while achieving favorable long-term outcomes (88). Regarding the duration of NET administration, most studies have been conducted over a period of 3–4 months, but current research suggests that up to 12 months of endocrine therapy is equally safe, but requires close monitoring (79, 87). Despite promising results, NET in ILC remains under-studied, and future trials should clarify the optimal treatment duration and define patient subgroups most likely to benefit.

#### 3.1.2 Surgery

##### 3.1.2.1 Breast surgery

The mastectomy rate in ILC exceeds that of IDC, likely due to its diffuse infiltration pattern and underestimation of tumor extent on imaging (61, 89–92). In recent years, however, the rate of breast-conserving therapy (BCT) in ILC has gradually increased (92), possibly related to the use of preoperative neoadjuvant chemotherapy (NACT), extended courses of neoadjuvant endocrine therapy (NET), and radiotherapy (83, 93). Multiple studies have demonstrated that BCT can achieve comparable local control in ILC, even in tumors greater than 4 cm, with no significant difference in local recurrence or long-term prognosis compared to IDC (94–96). Notably, compared with women treated with mastectomy in IDC cohorts, those undergoing BCT had significantly higher breast cancer-specific survival (BCSS) and a lower risk of breast cancer mortality, regardless of detection modality, prognostic features, or tumor subtype (97, 98).

While breast-conserving therapy (BCT) is increasingly applied in ILC, margin positivity remains a frequent concern. Studies have reported that more than half of ILC patients undergoing BCT present with positive margins after initial resection (99), and the risk of margin involvement is significantly higher than in IDC (89, 100). Lobular histology itself has been identified as an independent risk factor for margin positivity (101), which is clinically relevant given the two-fold increased risk of ipsilateral breast tumor recurrence associated with positive margins (102). These findings suggest that the choice of surgical procedure in ILC should be individualized, and that patients undergoing BCT should be counseled regarding the possibility of re-excision (103).

Interestingly, when only atypical lobular hyperplasia (ALH) or lobular carcinoma *in situ* (LCIS) is found at the surgical margin, reoperation is generally not recommended, as current evidence indicates a low progression rate and minimal impact on local recurrence risk (104, 105).

Given the high incidence and clinical implications of positive margins, predicting margin status preoperatively has become an important focus. Studies suggest that positive margins are more likely in patients with tumors ≥1.5 cm on mammography and in younger individuals (106, 107). Additionally, margin positivity risk should be considered preoperatively in cases with HR+/HER2− tumors, nonmass enhancement (NME) on MRI, or a histologic diagnosis of ILC (103). To improve local control in patients undergoing BCT and radiotherapy, intraoperative margin assessment and immediate re-excision have been beneficial—achieving negative margins in over one-quarter of patients during the initial surgery (108).

##### 3.1.2.2 Lymph node surgery

Axillary lymph node dissection (ALND) is more frequently performed in ILC compared to IDC, largely due to its higher lymph node (LN) stage, greater number of positive LNs, and a higher rate of occult metastasis (1). However, ALND is associated with considerable postoperative morbidity, including lymphedema and reduced shoulder mobility, which significantly impair quality of life (109). In recent years, sentinel lymph node biopsy (SLNB) has become the standard approach for axillary staging in LN-negative breast cancer (109, 110). For patients with 1–2 positive sentinel lymph nodes (SLNs) and no clinical axillary involvement, omitting ALND has shown equivalent survival outcomes, as demonstrated by the ACOSOG Z0011 trial. Supporting this, Wang et al. analyzed 1,269 ILC patients from the SEER database and reported similar prognostic outcomes between SLNB and ALND in selected patients with early-stage ILC following breast-conserving therapy (BCT) (111).

Nevertheless, in the neoadjuvant chemotherapy (NACT) setting, the accuracy of SLNB is substantially reduced due to altered lymphatic drainage, raising concerns about false-negative rates (FNR) (112). To address this, targeted axillary dissection (TAD) has been introduced since 2016. The NCCN guidelines recommend three technical criteria to maintain FNR below 10%: retrieval of pre-treatment marked nodes, dual tracer technique, and excision of at least three SLNs (113). Whitrock et al., using U.S. Cancer Database data, noted a growing adoption of TAD in node-positive (LN+) breast cancer patients after NACT from 2014 to 2017 (110).

**Figure 2:**
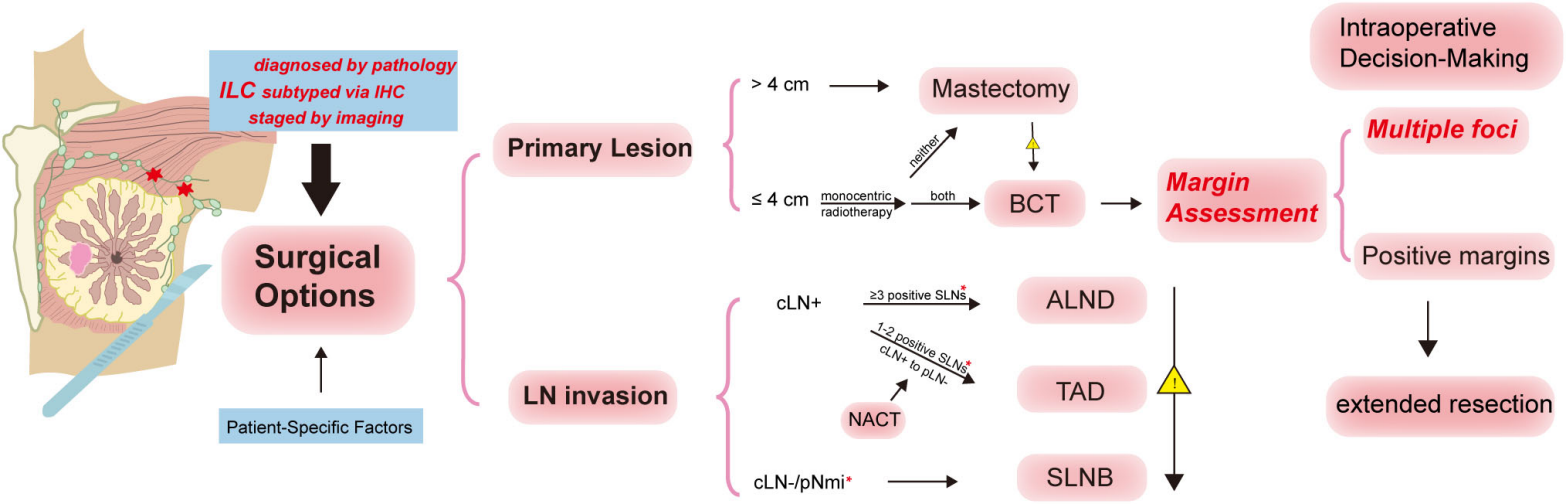
Surgical options for invasive lobular carcinoma (ILC). This figure summarizes the surgical decision-making process for ILC, including preoperative assessment, breast surgery and lymph node (LN) surgery and margin assessment. Because of the special characteristics of ILC, it is necessary to be more careful to make decisions of surgical degradation. For patients whose tumor size less than 4cm, monocentric lesion and eligible for radiotherapy, it is suggested to choose breast conserved therapy(BCT), while others are recommended to undergo mastectomy. * indicates that intraoperative frozen section (IFS) evaluation is required for margin assessment.

Despite these advances, ALND remains indispensable in certain clinical scenarios. Notably, TAD has not been validated in patients with persistently positive nodes after NACT (109, 110). Furthermore, ILC—particularly luminal A subtype—tends to present with ≥4 metastatic LNs and higher rates of non-sentinel node involvement compared to IDC, which increases the risk of under-staging when ALND is omitted (11, 114). These features underscore the need for cautious decision-making when considering de-escalation of axillary surgery in ILC. **Figure 2** illustrates the surgical decision-making process in ILC, incorporating preoperative evaluation, breast and lymph node surgery, and margin assessment. This visual guide emphasizes the need for caution in surgical de-escalation due to the unique characteristics of ILC.

#### 3.1.3 Adjuvant endocrine therapy

Most ILCs are positive for ER expression, leading the endocrine therapy the routine treatment for HR-positive early breast cancer. In the BIG 1–98 trial, letrozole was more efficacious than tamoxifen in early-stage ILC compared with early-stage IDC (115). In AI therapy, Weippl et al. compared the efficacy of anastrozole and exemestane in early-stage ILC and found that anastrozole had a better OS when compared to exemestane (116). Record et al. compared a treatment cohort of premenopausal women with aromatase inhibitors(AI) combined with ovarian function suppression(OFS)and tamoxifen combined with OFS and found that AIs significantly reduced the risk of death in premenopausal women (117). For early-stage ILC patients with high-risk features, adding CDK4/6 inhibitors to endocrine therapy has been proposed to reduce recurrence. A matched cohort study reported similar 3-year IDFS (86.9%) and DRFS (88.5%) for high-risk ILC compared to IDC, aligning with outcomes from the endocrine-alone arm of the monarchE trial (118). These findings suggest a potential benefit of CDK4/6 inhibition in ILC; however, since monarchE did not stratify by histology, this remains speculative. Prospective trials specifically targeting ILC are needed to validate this approach.

#### 3.1.4 Adjuvant chemotherapy

For ILC, the clinical utility of adjuvant chemotherapy (ACT) remains controversial due to its relatively limited efficacy. Multiple studies have reported that ACT confers less benefit in early-stage ILC compared to IDC (1). In a cohort of 520 ER+/HER2− ILC patients—379 of whom received chemotherapy—Öztekin et al. found no improvement in recurrence-free survival (RFS), breast cancer-specific survival (BCSS), or overall survival (OS) with the addition of chemotherapy to endocrine therapy, even in those with indications for ACT (119).

However, more recent studies suggest a nuanced view. Some data indicate that dose-dense chemotherapy may modestly reduce 10-year recurrence and mortality, with no significant difference between ILC and IDC when stratified by histology (120). In high-risk populations, chemotherapy appears to have a clearer benefit (120).

Regarding regimen selection, anthracyclines remain a standard component of ACT, though their cardiotoxicity raises concerns. Evidence from HR+/HER2− populations suggests that de-anthracycline regimens may offer comparable efficacy with improved tolerability (1). Nonetheless, a study by de Gregorio et al. reported that patients with pN2–N3 ILC still derive benefit from anthracycline-containing regimens (121), indicating that complete omission of anthracyclines in high-risk ILC should be approached with caution.

Critically, most studies lack ILC-specific stratification or were retrospective in nature, limiting their applicability to this histologic subtype. While emerging data support a more individualized approach, current treatment decisions are still largely guided by clinicopathological features rather than robust ILC-specific evidence. Therefore, future prospective trials are warranted to clarify the optimal role and regimen of ACT in ILC.

### 3.2 Treatment of advanced and metastatic ILC

#### 3.2.1 Endocrine therapy combined with CDK4/6 inhibitors in advanced ILC

CDK4/6 inhibitors combined with endocrine therapy has become the first-line treatment for advanced ER positive ILC. In recent years, the addition of CDK4/6 inhibitors to endocrine regimens has redefined the first-line treatment landscape, with agents such as Palbociclib, Ribociclib, Abemaciclib, and Dalpiciclib approved or under investigation.Although all CDK4/6 inhibitors share a common target, they differ in pharmacologic properties, dosing schedules, and toxicity profiles. For instance, Abemaciclib is administered continuously and has greater single-agent activity, while Ribociclib is associated with more prominent QTc prolongation and Palbociclib is more commonly linked to neutropenia.

Despite the large representation of HR+ patients in pivotal CDK4/6 inhibitor trials (MONALEESA, MONARCH, PALOMA series), ILC-specific outcomes remain underreported. *Post hoc* analyses of MONARCH and PALOMA trials did include lobular subsets, but sample sizes were small, and no histology-stratified efficacy conclusions could be firmly drawn. This is a major evidence gap, given the biological and clinical distinctiveness of ILC.

Anecdotal reports suggest promising activity of CDK4/6 inhibitors in ILC. Nuno Rodrigues Alves et al. reported a case of an ILC patient who developed bilateral orbital metastases and achieved significant efficacy after treatment with letrozole in combination with Abemaciclib (21). Similarly, Gao et al. reported a case of ILC with initial bone metastases who later developed peritoneal progression during tamoxifen plus Abemaciclib treatment; subsequent genomic analysis revealed ESR1 and PIK3CA mutations (122). While mechanistic interpretation was beyond the report’s scope, such cases underscore the real-world application of CDK4/6 inhibition in ILC and the clinical complexity encountered in treatment-refractory settings.

#### 3.2.2 Chemotherapy

For patients with advanced invasive lobular carcinoma (ILC), chemotherapy is generally reserved for endocrine-resistant disease or visceral crisis situations (1). A study has demonstrated that single-agent capecitabine(CAP) have a superior progression-free survival(PFS) to taxanes(TAX) in ET-refractory HR+ HER2-negative mILC, showing the potential benefit of CAP in this patient subgroup (123). Eribulin appears to offer comparable efficacy in metastatic ILC and IDC patients following prior treatment with anthracyclines and taxanes, as reported by Pérez-Garcia et al. (124). Prospective, ILC-enriched cohorts are urgently needed to validate these regimens. Moreover, single-agent regimens are generally better tolerated and may be preferable in patients with comorbidities or those with indolent but progressing disease, particularly when aiming to stabilize visceral involvement while minimizing toxicity.

### 3.3 Targetable molecular and immune pathways in ILC

**Figure 3:**
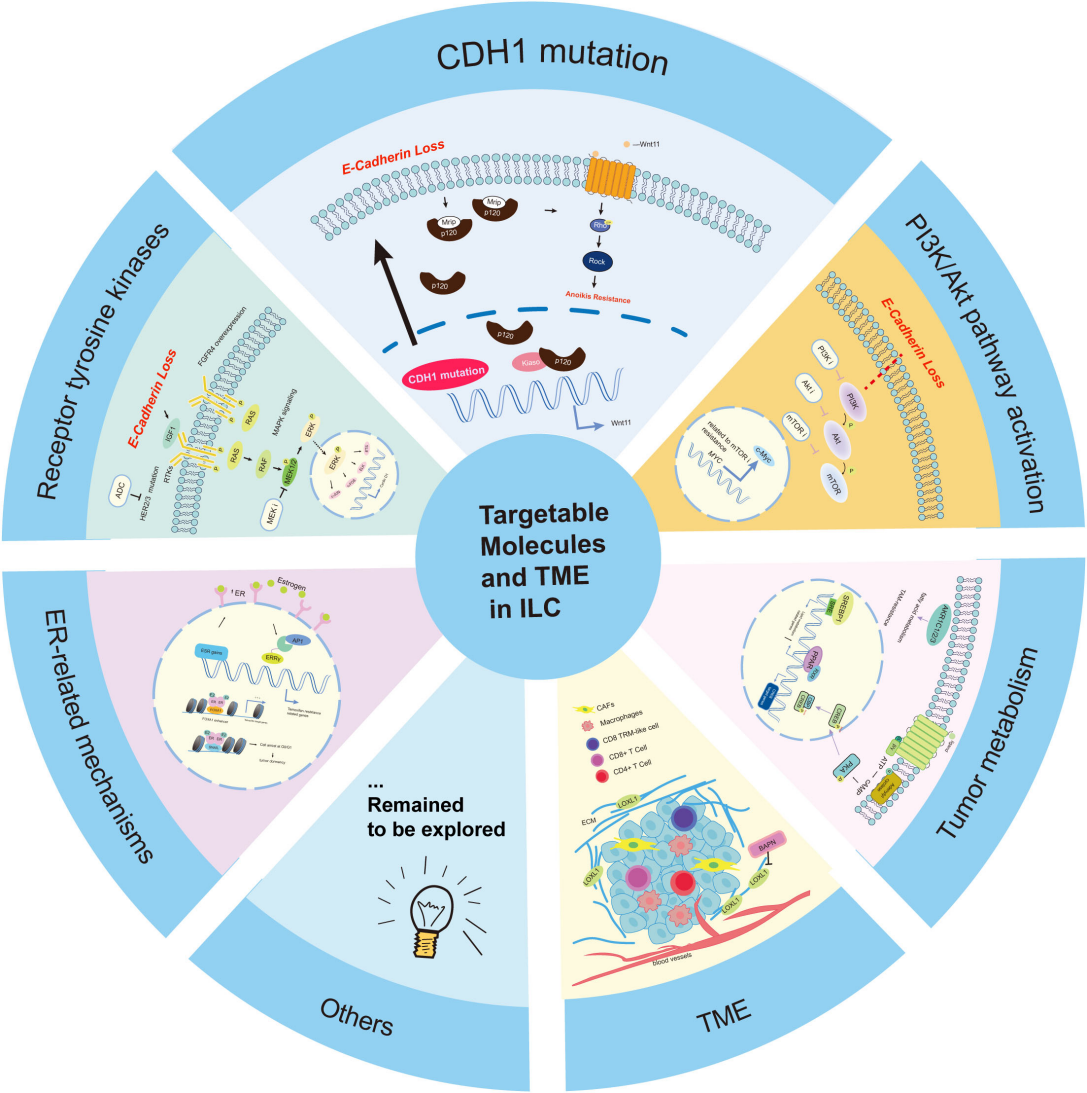
Targetable molecules and TME(tumor microenvironment) in invasive lobular carcinoma (ILC). This figure summarizes targetable molecular and immune pathways in ILC. CDH1 loss and E-cadherin dysfunction may be associated with alterations in downstream pathways such as PI3K/Akt/mTOR and IGF1R, although the causality remains unclear. Common genetic alterations include PIK3CA, PTEN loss, and receptor tyrosine kinase mutations (e.g., ERBB2, FGFR4). ER-related adaptations and immune evasion also contribute to therapy resistance. These pathways represent areas of ongoing investigation and may support future precision therapy development.

Despite the generally favorable response of HR-positive ILC to endocrine therapy, acquired resistance remains a major clinical obstacle. Hanker et al. comprehensively summarized resistance mechanisms in HR+ advanced breast cancer, encompassing somatic mutations, transcription factor dysregulation, DNA repair deficiencies, epigenetic alterations, coactivator overexpression, epithelial–mesenchymal transition (EMT), stem cell–like phenotypes, metabolic shifts, and modifications in the tumor microenvironment (TME) (125). However, the molecular landscape underlying endocrine resistance in ILC differs substantially from that in invasive ductal carcinoma (IDC), including distinctive somatic mutation patterns, ER-regulatory transcriptomes, and expression profiles (125, 126). These differences suggest ILC may rely on unique molecular vulnerabilities beyond conventional mechanisms. **Figure 3** summarizes the targetable molecular and immune pathways implicated in ILC, including potential mechanisms of endocrine resistance and novel therapeutic opportunities that may guide future precision treatments.

#### 3.3.1 CDH1 mutation and E-cadherin loss

In ILC, the loss of E-cadherin (E-Ca) resulting from CDH1 mutations represents a defining molecular feature. As summarized by Sijnesael et al., E-Ca loss leads to intracellular translocation of p120-catenin (p120), which binds to the zinc finger transcription factor Kaiso, thereby relieving Kaiso-mediated transcriptional repression at cKBS (Kaiso-binding site) motifs (127). This derepression may activate non-canonical Wnt signaling, particularly Wnt11, contributing to Rho pathway activation, cytoskeletal remodeling, and a loss of anoikis—a potential mechanism contributing to endocrine resistance in ILC (127, 128). Furthermore, Sikora et al. identified the Wnt4 pathway as another candidate driver of endocrine resistance in lobular models (129). While these findings offer intriguing mechanistic insights, they are primarily derived from preclinical models, and their relevance to patient outcomes remains to be fully validated. Notably, the causal link between Wnt signaling activation and therapeutic resistance in ILC has not yet been firmly established, and further functional and clinical studies are warranted to clarify these relationships.

#### 3.3.2 Signal transduction pathways in endocrine resistance

##### 3.3.2.1 PI3K/AKT/mTOR pathway and its resistance bypass mechanisms

Activation of the PI3K/AKT/mTOR pathway is commonly seen in ILC, often due to PIK3CA mutations or PTEN loss (130). Boelens et al. demonstrated that dual inhibition using the PI3K/mTOR inhibitor BEZ235 exhibited antitumor effects in ILC models (130). However, the clinical value of PIK3CA mutations remains controversial: they occur early in lobular tumorigenesis and may not necessarily indicate aggressive disease (131), with some studies even linking them to a reduced risk of local relapse (132). Conversely, PTEN deletions correlate with significantly shorter time to progression (131), highlighting that not all alterations in this pathway carry equal prognostic weight. Moreover, resistance to mTOR inhibitors (mTORi) has been linked to MYC activation, which counteracts mTORi-induced translational suppression by upregulating ribosomal protein translation (133). This mechanism, uncovered in a multi-omics analysis of mouse ILCs, illustrates the need to account for feedback and bypass loops when targeting this pathway. Overall, while PI3K pathway inhibitors show promise, patient stratification based on molecular context remains a challenge.

##### 3.3.2.2 RTKs and alternative targetable pathways in endocrine resistance

In addition to the PI3K pathway, alterations in multiple receptor tyrosine kinases (RTKs) have been identified in ILC. HER2 mutations have been associated with reduced endocrine responsiveness in early-stage ILC, and emerging evidence suggests that targeting HER2 mutations could improve outcomes (134, 135). HER3 mutations are increasingly reported, yet the efficacy of anti-HER3 therapy remains unclear. A distinct target is FGFR4, found to be overexpressed in endocrine-resistant ILC cell lines and metastatic ER+ ILC samples (136). FGFR4 mutations were also detected in patient samples, suggesting its involvement in acquired resistance (136). Another potentially actionable axis is the IGF1R pathway. In E-cadherin-deficient ILC, loss of CDH1 was found to activate IGF1R signaling, increasing sensitivity to IGF1R/InsR-targeted therapies (137). This interaction highlights the interplay between cell adhesion loss and growth factor signaling. Moreover, mutations in metabotropic glutamate receptors (GRMs or mGluRs) were found to upregulate MAPK signaling and impair tamoxifen sensitivity (138). Preclinical data support the use of MEK inhibitors or glutamate-release blockers to restore endocrine response in this context (139). However, these findings are mostly based on non-clinical studies and require further validation in human ILC-specific cohorts.

#### 3.3.3 Hormone receptor-related alterations

Endocrine resistance in ILC is increasingly understood to involve distinct estrogen receptor (ER)-related mechanisms. A prominent model proposed by Nardone et al. highlights the unique chromatin organization of ILC, which facilitates the binding of FOXA1 and ER to super-enhancers (140). This process helps sustain ER transcriptional activity even under tamoxifen (TAM) treatment, thus driving resistance despite the drug’s presence (140). While this mechanism sheds light on chromatin-based resistance pathways, it remains largely correlative, and functional studies directly disrupting enhancer-ER interactions in ILC are still limited.

Transcriptional reprogramming downstream of ER also appears to play a role. For example, the zinc finger transcription factor SNAIL, a well-known regulator of epithelial-mesenchymal transition (EMT) and a target gene of ER, was shown by Bossart et al. to be upregulated when both TAM and estrogen are present. ER recruitment to the SNAIL promoter was enhanced, and SNAIL overexpression was associated with G0/G1 arrest and reduced proliferation, implying that it may induce a dormant state in ILC cells that confers resistance (141). This proposed dormancy model, while intriguing, remains speculative. Whether SNAIL-mediated quiescence is a reversible survival mechanism or contributes to true therapy escape is yet to be validated *in vivo*.

Genomic alterations of the ER itself also contribute. ESR1 gene amplification (ESR1 gains) has been reported in over 20% of ILC samples, potentially enhancing ligand-independent ER signaling (126). Furthermore, Rebecca B. Riggins et al. identified the ERRγ/AP1 signaling axis as a novel contributor to TAM resistance, where ERRγ expression was associated with poor treatment response (142). However, ERRγ’s functional role in lobular carcinoma remains undercharacterized, and current evidence is mostly based on expression data without mechanistic dissection in ILC-specific contexts.

Taken together, these studies collectively support a multifaceted model of endocrine resistance in ILC involving structural enhancer rewiring, transcriptional reprogramming, and receptor-level genetic changes. Nevertheless, most findings are extrapolated from preclinical or mixed histology models. Future ILC-specific functional validation will be crucial to determine the translational value of these mechanisms.

#### 3.3.4 Distinct lipid metabolism pathways

Alterations in lipid metabolism have been increasingly implicated in endocrine resistance in ILC, particularly in response to tamoxifen (TAM) and aromatase inhibitors (AIs). Sivadas et al. identified significant enrichment of peroxisome proliferator-activated receptor (PPAR) signaling and pathways regulating lipolysis in adipocytes within ILC tumors, suggesting a metabolic phenotype distinct from that of invasive ductal carcinoma (IDC) (143). Additionally, Narayanan et al., through integrated analysis of SCAN-B, TCGA, and METABRIC datasets, reported that cAMP/PKA/CREB signaling activity was elevated in ILC relative to IDC (144). This finding was further validated in both cell line and organoid models, supporting the notion that ILC exhibits a unique metabolic-transcriptional axis (144).

Further mechanistic insight was provided by Xu et al., who demonstrated that TAM-resistant ILC cells exhibited upregulated expression of aldo-keto reductase family members (AKR1C1/2/3), enzymes involved in fatty acid metabolism (145). Knockdown of these genes restored TAM sensitivity, implicating them in a functional resistance mechanism (145). Similarly, in the context of AI resistance, elevated expression of sterol regulatory element-binding protein 1 (SREBP1)—a master regulator of lipid and cholesterol biosynthesis—was correlated with lack of clinical response, highlighting a potential link between *de novo* lipogenesis and therapeutic escape in ILC (146).

Although these findings suggest compelling associations between lipid metabolic reprogramming and endocrine resistance in ILC, most current evidence is derived from transcriptomic analyses or *in vitro* systems. The clinical relevance and causality of these pathways remain to be validated in prospective patient cohorts and interventional studies. Moreover, whether targeting metabolic dependencies could effectively reverse resistance or enhance endocrine response in ILC has not yet been conclusively demonstrated. Therefore, while these metabolic signatures offer promising avenues for further exploration, they should currently be interpreted as hypothesis-generating rather than practice-changing.

#### 3.3.5 DNA damage response and repair pathways

DNA repair mechanisms are known to play a crucial role in determining both therapeutic sensitivity and resistance in breast cancer. In a comprehensive transcriptomic analysis, Mohamed et al. identified a broad spectrum of DNA repair genes involved in diverse pathways, including translesion DNA synthesis, double-strand break (DSB) repair, homology-directed repair (HDR), Fanconi anemia (FA), and base excision repair (BER), among others (147). Interestingly, while ILC and IDC displayed broadly similar levels of DNA repair gene expression, their underlying transcriptomic signatures were notably distinct (125). While this observation suggests the core components of DNA repair machinery are conserved between subtypes, the transcriptional divergence may reflect subtype-specific regulatory contexts or functional consequences. Notably, current analyses rely on bulk data, potentially masking cell-type–specific activity or microenvironmental modulation. Thus, whether DNA repair contributes meaningfully to resistance or therapeutic targeting in ILC remains unresolved and warrants further functional investigation.

#### 3.3.6 TME (tumor microenvironment) and immune modulation

The tumor microenvironment (TME) plays a crucial role in regulating tumor growth, metabolism, immune evasion, and therapeutic response in ILC. Compared to IDC, ILC exhibits a distinct stromal and immune landscape that may inform novel treatment strategies.

##### 3.3.6.1 Stromal remodeling and cancer-associated fibroblasts (CAFs)

ILC is characterized by a more abundant but less mature vasculature and enriched stromal components, including CAFs and altered extracellular matrix (ECM) composition (148). CAFs in ILC differ from those in IDC, with higher expression of markers such as FAP-α and FSP-1/S100A4 (149). These cells are genetically stable yet epigenetically reprogrammed, contributing persistently to tumor progression (149).

Mechanistically, ILC CAFs secrete pregnancy-associated plasma protein A (PAPP-A), enhancing IGF-1 bioavailability and activating the IGF1R pathway in tumor epithelial cells. This paracrine loop may not only promote growth and invasion but also create an immunosuppressive microenvironment by affecting immune cell trafficking (150). These findings support a potential CAF-driven IGF1/IGF1R paracrine loop in ILC; however, direct links to immune suppression or metastatic progression remain to be functionally validated. Notably, Sflomos et al. identified overexpression of ECM regulators including LOXL1 in ILC stroma; inhibition of LOXL1 via BAPN disrupted ECM integrity, reduced ER signaling, and attenuated tumor progression (151), offering a promising CAF-targeted strategy. Recent spatial analyses revealed subtype-specific CAF profiles: pleomorphic ILCs were enriched in FAP+ and S100A4+ CAFs, while α-SMA+ CAFs were more common and spatially proximal in classic ILCs (152). This highlights the heterogeneity of CAFs within ILC and supports the rationale for subtype-specific stromal targeting.

##### 3.3.6.2 Immune landscape and therapeutic implications

ILC demonstrates a predominantly “immune cold” phenotype: reduced TILs, increased TAMs, and a higher M2:M1 macrophage ratio—particularly in the stromal compartment—contribute to an immune-excluded microenvironment (153). Spatial dislocation of immune cells from tumor nests further impairs cytotoxic engagement and likely underlies the limited efficacy of immune checkpoint inhibitors (ICIs) in ILC (153). Nevertheless, about 17% of ILC tumors express PD-L1 on tumor cells or infiltrating lymphocytes, with a corresponding enrichment of CD8+ T cells, suggesting a potentially responsive subgroup (154). The GELATO trial reported a 20% response rate to carboplatin and PD-L1 blockade in triple-negative ILC (TN-ILC), a rare and underexplored subset with possibly greater immunogenicity. However, the limited sample size precludes firm conclusions and underscores the need for larger, subtype-specific trials.

##### 3.3.6.3 Future directions and therapeutic opportunities

Collectively, these findings reveal a complex interplay between CAFs, ECM remodeling, and immune evasion in ILC. The CAF–IGF axis and LOXL1-mediated ECM stiffening represent promising therapeutic targets. Moreover, integrating CAF-targeted agents or macrophage modulators (e.g., HDAC inhibitors) with ICIs may help overcome immune exclusion and resistance in ILC. To this end, single-cell and spatial transcriptomic approaches are urgently needed to delineate stromal-immune crosstalk and identify actionable stromal subtypes in ILC.

## 4 Discussion

Invasive lobular carcinoma (ILC) displays distinct metastatic patterns compared to invasive ductal carcinoma (IDC), with a predisposition for the peritoneum, retroperitoneum, gastrointestinal tract, and leptomeninges—sites often missed due to subtle radiologic features and low FDG avidity (155, 156). Alternative modalities such as contrast-enhanced CT, MRI, or CSF cytology should be considered in high-risk patients to avoid disease underestimation. While therapeutic options for such metastases remain limited, experimental strategies like hyperthermic intraperitoneal chemotherapy (HIPEC) or intrathecal chemotherapy merit further exploration. Our analysis suggests that different ILC histologic subtypes may demonstrate variable metastatic propensities. However, histology-based stratification alone may not fully capture metastatic risk. Clinical parameters such as tumor burden, nodal status, anatomic involvement, and Ki-67 index should be integrated to refine treatment planning. Similarly, molecular subtypes may reflect intrinsic biology but lack consistent correlation with specific metastatic sites, due to limited and retrospective datasets. The scarcity of rare histologic subtypes and underutilization of detailed subtype diagnosis in earlier decades have contributed to data insufficiency. Artificial intelligence (AI) holds promise in addressing these gaps. Trained models can identify CDH1 inactivation—a hallmark of ILC—and assist in understanding its downstream effects, including altered metastatic routes and immune exclusion (157, 158). AI can also enhance detection of occult nodal metastases, improving staging accuracy and informing systemic therapy choices (159). Furthermore, AI-driven pathology platforms may aid in building more precise classification systems, incorporating multi-modal data to better define metastatic risk and optimize treatment timing, particularly for chemotherapy in ILC. Current stratification strategies remain largely hypothesis-generating. Prospective multi-institutional cohorts, coupled with spatial profiling and AI-driven analytics, are needed to validate how histologic, molecular, and clinical features jointly determine ILC metastatic behavior and treatment response.

**Figure 4:**
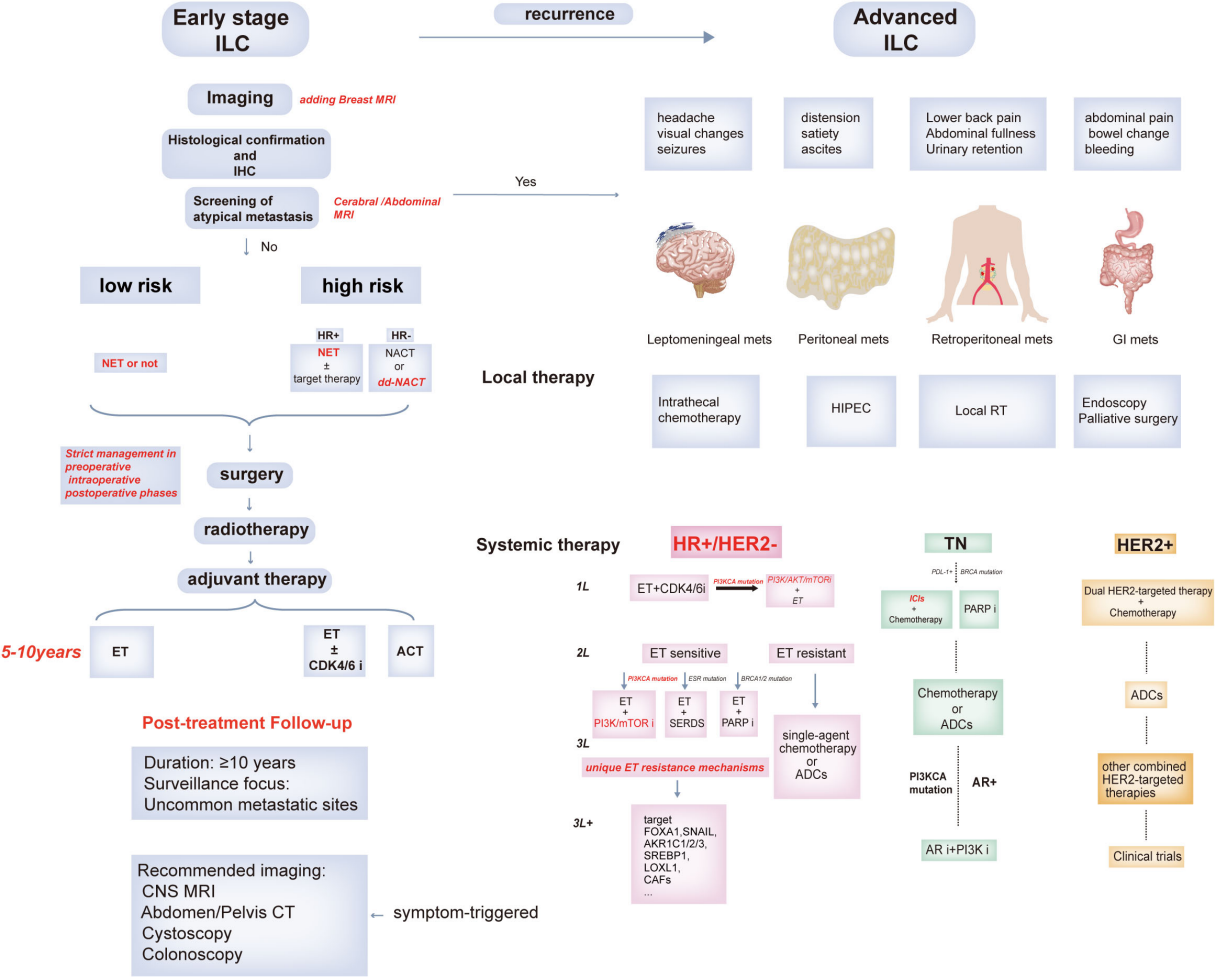
Conceptual framework for stage-specific management in invasive lobular carcinoma (ILC). This schematic illustrates a proposed treatment framework for ILC, integrating current clinical practice with emerging stratification strategies. In early-stage disease, accurate diagnosis is followed by risk-adapted treatment selection: Low-risk patients may undergo surgery alone or receive neoadjuvant endocrine therapy. High-risk patients, based on molecular subtypes, pathological characteristics and tumor stages, may benefit from neoadjuvant chemotherapy or intensified endocrine regimens. Postoperative high-risk individuals may consider adjuvant CDK4/6 inhibitors or chemotherapy intensification. Extended follow-up is recommended, with symptom-guided evaluation for atypical metastatic sites such as the peritoneum, gastrointestinal tract, and central nervous system. In advanced-stage ILC, local and systemic therapies should proceed concurrently. Systemic therapy decisions should incorporate molecular subtype and ILC-specific alterations (e.g., CDH1 loss, PIK3CA mutations, rare actionable targets).This framework remains exploratory and hypothesis-generating, underscoring the need for prospective data and ILC-specific clinical trials to refine precision treatment approaches.

Early-stage ILC treatment remains largely empirical, with limited evidence supporting chemotherapy efficacy. Neoadjuvant chemotherapy (NACT) often yields suboptimal response in ILC, while the role of adjuvant chemotherapy (ACT) remains controversial. In contrast, neoadjuvant endocrine therapy (NET) is gaining attention but lacks definitive clinical guidance or routine application. Given these uncertainties, our review advocates a dual-layered stratification incorporating both molecular profiles (e.g., luminal A/B) and clinical-pathologic parameters (tumor size, nodal status, Ki-67 index, histologic subtype) to define high-risk vs. low-risk patients. Such integrative assessment may better inform surgical planning and adjuvant treatment strategies. For advanced disease, systemic therapy guided by hormone receptor (HR) and HER2 status remains the cornerstone. However, local treatment options may offer added benefit, especially for symptom-driven metastatic lesions: HIPEC for peritoneal carcinomatosis, intrathecal chemotherapy for leptomeningeal disease, or stereotactic radiotherapy for oligometastatic involvement. Targeted therapies aimed at frequent ILC alterations—CDH1 loss, PI3KCA mutations, ERBB2 mutations—may further refine systemic approaches (160). Meanwhile, immune checkpoint blockade and other novel modalities are under exploration, though their efficacy in ILC remains to be fully established. **Figure 4** outlines the current and emerging treatment paradigms for ILC, integrating histologic subtypes, endocrine sensitivity, and molecular vulnerabilities to support stage-specific therapeutic strategies.

To refine treatment strategies, recent trials have introduced multi-omics-based classification systems, notably the Subtype Network Fusion (SNF) framework developed by Professor Zhimin Shao’s group. Originally applied to IDC, SNF stratifies luminal A tumors into four transcriptomic subtypes (SNF1–SNF4), each associated with unique therapeutic vulnerabilities (161). Between 2022 and 2024, three early-stage HR+/HER2− trials have adopted SNF-guided interventions—for example, NCT05891093 evaluates fluzoparib in SNF3, while NCT05889871 investigates apatinib in SNF4 tumors. Though SNF has yet to be validated in ILC, it provides a model for biologically informed treatment selection. Beyond SNF, several ILC-specific trials have emerged. These include ROS1 inhibitor repotrectinib in metastatic ILC (NCT04551495), HER2 mutation-targeted therapies (NCT05911910), and small molecule TKIs in the advanced setting (NCT06408168). In early-stage HR+ ILC, NCT06144268 explores CDK4/6 inhibitors in the neoadjuvant setting. Additionally, NCT06067503 investigates predictive biomarkers associated with endocrine resistance specifically in ILC. **Table 1** compiles recent (2022–2024) clinical trials specific to ILC, reflecting ongoing progress in endocrine and targeted therapies. **Table 2** provides a parallel summary of clinical trials in early-stage HR+/HER2− breast cancer, emphasizing SNF subtypes and novel molecular targets.

**Table 1:** On-going clinical trials of invasive lobular carcinoma from 2022-2024.

Serial number	Title	Stage	Start time	End time	Phase	Number of patients	Arms	Primary endpoint:
NCT04551495	Neoadjuvant Study of Targeting ROS1 in Combination With Endocrine Therapy in Invasive Lobular Carcinoma of the Breast	early	2021/1/14	2025/1/1	2	65	single arm: Subjects will receive four 28-day cycles of letrozole 2.5 mg daily in combination with neratinib 600 mg daily. Pre-menopausal women will receive goserelin 3.6 mg every 28 days.	Residual Cancer Burden (RCB)
NCT0591910	Neoadjuvant Neratinib in Stage I-III HER2-Mutated Lobular Breast Cancers	early	2025/1/31	2031/4/30	2	30	4weeks as a cycle, for 24 weeks Treatment A (endocrine therapy) Treatment B (endocrine therapy, neratinib)	Preoperative endocrine prognostic index score(Up to 5 years)
NCT02764541	Palbociclib and Endocrine Therapy for Lobular Breast Cancer Preoperative Study (PELOPS): A Randomized Phase II Study of Palbociclib With Letrozole Versus Letrozole Alone for Invasive Lobular Carcinoma and Invasive Ductal Carcinoma (PELOPS)	early	2016/5/24	2031/4/1	2	195(84 43.8%)	Arm1:Tamoxifen–endocrine therapy Arm2: letrozole–endocrine therapy Arm3: Tamoxifen–Palbociclib+endocrine therapy Arm4: letrozole–Palbociclib+endocrine therapy	Difference in Anti-proliferative Activity of Patients Given Letrozole Versus Tamoxifen During the Window Phase; Pathologic Complete Response (pCR) of Patients Given Endocrine Therapy Plus Palbociclib and of Endocrine Therapy Alone During the Treatment Phase
NCT01953588	Fulvestrant and/​or Anastrozole in Treating Postmenopausal Patients With Stage II-III Breast Cancer Undergoing Surgery	early	2013/12/13	2025/8/31	3	1473	Arm I (anastrozole); Arm II (fulvestrant); Arm III (anastrozole and fulvestrant)	Rate of endocrine resistant disease-(First Phase) [Time Frame: Up to 24 weeks] Pathologic complete response rate-(pCR rate) [Time Frame: Up to 24 weeks] Recurrence-free survival (RFS)-(Second Phase) [Time Frame: Up to 5 years]
NCT06408168	Phase II Study of REPotrectinib With or Without Fulvestrant in Patients With Hormone Receptor-positive Human Epidermal Growth Factor 2-negative Metastatic Invasive Lobular Carcinoma Who Received a Prior Endocrine Therapy in Combination With Cyclin-dependent Kinase 4 and 6 Inhibitor (REPLOT Trial) CDK4/6	advanced	2024/8/8	2027/12/31	1, 2	58	Cohort 1:fulvestrant combined with repotrectinib (either use at the same time or in a sequential way in every treatment cycle for 6 months) Cohort 2: have received fulcestrant in the earlier treatment and will receive repotreatinib alone	6-month progression free survival (PFS)
NCT06067503	Integrating Minimally Invasive Biomarkers of Estrogen Signaling to Detect Endocrine Therapy Resistance in Metastatic Invasive Lobular Breast Cancer	advanced	2024/4/30	2026/1/1	2	8(recruiting)	Experimental: Participants with ER/PR+ metastatic lobular breast cancer (LBC)	1.Number of Participants who have decreased FFNP uptake on PET/CT in response to endocrine therapy 2.Number of Participants who have decrease in circulating tumor cell estrogen signaling in response to endocrine therapy 3.Baseline level and on-treatment CTC ESR1 and estrogen regulated gene expression will be evaluated as well as endocrine-resistance associated mutations including ESR1 (though rare in this patient population) and PGR. Number of Participants who have a decrease in concentration of Circulating Tumor DNA in response to endocrine therapy

**Table 2:** On-going clinical trials of early stage HR-positive HER2-negative Breast Carcinoma from 2022-2024.

Serial number	Name	Start time	End time	Phase	Number of patients	Inclusion criteria	Arms	Primary endpoint
NCT05809024	Select the Appropriate Population for Adding CDK4/6i to Neoadjuvant Endocrine Therapy in High-Risk Early HR+/HER2-breast Cancer Based on Molecular Marker CDK4/6	2023/3/1	2026/3/25	4	100	cT2-cT4/cN0-cN3/cM0 (clinical phase II and III);HR+/HER2- breast cancer	Single Arm:Hormone receptor positive,HER2 negative participants will receive letrozole as neoadjuvant endocrine therapy, if ki67 was higher than 10% after two weeks,CDK4/6 Inhibitor was added.	ki67 index [Time Frame: Up to approximately 2 weeks]
NCT05512416	Neoadjuvant Dalpiciclib Plus Letrozole for HR+/HER2- Breast Cancer: A Single Arm, Open Label, Phase II Trial	2022/8/1	2024/5/1	2	35	Operable patients with ER+ (>10%), HER2- invasive breast carcinomas, regardless of PR level. ;	Single Arm:patients with stage IIB-III HR+/HER2- breast cancer;Six 4-week cycles of dalpiciclib orally, 150mg, day 1-21, and letrozole orally, 2.5 mg, day 1-28	Complete cell-cycle arrest at C1D15, defined as ki67 ≤ 2.7% [Time Frame: up 2 years] From the date into this study(signed ICF) to C1D15,defined as ki67 ≤ 2.7%
NCT05512780	An Exploratory Clinical Study of CDK4/6 Inhibitor Dalpiciclib Combined With Letrozole in Neoadjuvant Treatment of Stage II-III HR-positive/HER2-negative Breast Cancer	2022/9/10	2024/8/10	2	30	Treatment-naive patients with (ER) positive (>10%), HER2 -negative invasive breast cancer regardless of PR expression level.	Single Arm:Dalpiciclib combined with Letrozole,28 days as one cycle. Dalpiciclib: 150 mg (p.o.) was given once daily for 3 weeks, followed by 1 week off in each 4-week cycle. Letrozole: 2.5mg, p.o., once a day, continuous administration.	ORR [Time Frame: 24 months] Objective response rate
NCT06650748	Multigene Risk Score Combined With Ki-67 Dynamic Assessment in Stratified Neoadjuvant Endocrine Therapy Treatment With or Without CDK4/6 Inhibitors in HR+/HER2- Breast Cancer: a Randomize-controlled Study	2024/11/15	2028/4/1	2	100	≤T2N1M0 HR+/HER2- invasive breast cancer, Ki67≥+20%, ER expression >50%	Arm1:gene high-risk according to Epclin who are insensitive to single-agent AI treatment for two weeks; Arm2:gene high-risk according to Epclin who are sensitive to single-agent AI treatment for two weeks and gene low-risk according to Epclin who are insensitive to single-agent AI treatment for two weeks, randomly assigned to letrozole treatment arm; Arm3:gene high-risk according to Epclin who are sensitive to single-agent AI treatment for two weeks and gene low-risk according to Epclin who are insensitive to single-agent AI treatment for two weeks, randomly assigned to Dalpiciclib and letrozole treatment arm; Arm4:gene low-risk according to Epclin who are sensitive to single-agent AI treatment for two weeks	The proportion of (PEPI score 0 + pCR) in patients with discordant EPclin scores and Ki67 assessments who were randomly assigned to the “+CDK4/6i” group [Time Frame: Start of treatment until 6-month follow-up]
NCT05891093	A Prospective, Randomized, Open-label Phase III Clinical Study of the Efficacy and Safety of Fluzoparib Combined With Adjuvant Endocrine Therapy Versus Adjuvant Endocrine Therapy for HR+/HER2- SNF3-subtype Early Breast Cancer (BCTOP-L-A01)	2023/6/1	2031/5/31	1	766	stage T2-4N0-3M0, ER+/HER2- ;SNF3;have previously received neoadjuvant chemotherapy and/or adjuvant chemotherapy;	Experimental: Fluzoparib+Endocrine Therapy Fluzoparib 50mg bid orally for 1 year, combined with physician’s choice of endocrine therapy as clinically indicated (eg, aromatase inhibitor, tamoxifen, toremifene endocrine therapy for 5 to 10 years; CDK4/6 inhibitor therapy for 2 years; ovarian function suppression with LHRH agonist). Active Comparator: Endocrine Therapy Physician’s choice of endocrine therapy as clinically indicated (eg, aromatase inhibitor, tamoxifen, toremifene endocrine therapy for 5 to 10 years; CDK4/6 inhibitor therapy for 2 years; ovarian function suppression with LHRH agonist).	invasive disease free survival (iDFS)
NCT05889871	A Randomized, Controlled, Open-label , Phase III Clinical Trial of Adjuvant Intensive Therapy for HR+/HER2-SNF4 Early Breast Cancer Based on SNF Molecular Classification	2023/6/1	2026/6/1	3	916	pT2-4N0-3M0 HR+/HER2- invasive breast cancer ;SNF4 subtype;No more than 16 months from surgery to randomization, and no more than 12 weeks after non-endocrine therapy;	Experimental: Standard endocrine therapy plus Apatinib 5 to 10 years of endocrine therapy (e.g., aromatase inhibitors, tamoxifen, LHRH agonists, etc.) and 2 years of CDK4/6 inhibitors, depending on clinical indications. plus Apatinib, 250mg orally once a day; Active Comparator: Standard endocrine therapy 5 to 10 years of endocrine therapy (e.g., aromatase inhibitors, tamoxifen, LHRH agonists, etc.) and 2 years of CDK4/6 inhibitors, depending on clinical indications.	3-year survival without invasive disease (iDFS)
NCT06650423	ONCO-ADHER: Adherence to Treatment With an Aromatase Inhibitors With or Without Abemaciclib in Patients With Early-stage, Endocrine-dependent, HER-2-negative Breast Cancer	2024/10/20	2026/3/1	Observational	319	Early HR+/HER-2- BC;Patient is receiving adjuvant therapy with an aromatase inhibitor (letrozole, anastrozole or exemestane), with or without a CDK4/6 inhibitor abemaciclib, for no more than 18 months,	Arm1:Aromatase inhibitor + abemaciclib Adult women with early HR+ HER2- breast cancer, eligible for treatment with aromatase inhibitor + abemaciclib, both prescribed prior inclusion into study, irrespective of protocol, as per regular clinical practice; Arm2:Aromatase inhibitor Adult women with early HR+ HER2- breast cancer, eligible for treatment with aromatase inhibitor, prescribed prior inclusion into study, irrespective of protocol, as per regular clinical practice	Medication adherence in proportion of days covered (PDC); Medication adherence in proportion of days covered (PDC) [Time Frame: Month 6 after starting dose]

## 5 Conclusion

This review provides a comprehensive analysis of invasive lobular carcinoma (ILC) by examining metastatic patterns across different histologic subtypes, alongside a preliminary exploration of subtype-based risk stratification. While current evidence for formal risk categories remains limited, integrating histologic and molecular features offers insights into the broader metastatic spectrum of ILC compared with invasive ductal carcinoma (IDC). In addition, we summarize current clinical management strategies and highlight potential avenues for targeted and immunotherapeutic approaches. Our review of recent (2022–2024) clinical trials reveals growing interest in ILC-specific targets and paves the way for individualized treatment pathways. Overall, this work emphasizes the unique metastatic behavior of ILC as a foundation for guiding clinical decision-making, while underscoring the need for large-scale studies, multi-omics integration, and prospective trials to refine personalized treatment pathways.

## Ethics statement

The study was conducted according to the guidelines of the Declaration of Helsinki, approved by the Institutional Review Board (IRB) at the First Affiliated Hospital of Xi’an Jiaotong University (The approval number: XJTU1AF2025LSYY-458).

## Glossary

ILC: Invasive lobular carcinoma
IDC: Invasive ductal carcinoma
NACT: Neoadjuvant chemotherapy
NET: Neoadjuvant endocrine therapy
HR: Hormone-receptor
LN: Lymph node
PLC: Pleomorphic lobular carcinoma
cILC: Classic invasive lobular carcinoma
ER: Estrogen receptor
HER-2: Human epidermal growth factor receptor 2
TN: Triple negative
mIDLC: Mixed invasive ductal lobular carcinoma
PR: Progesterone receptor
AR: Androgen receptor
pCR: Pathological complete response
iddEPC: Intense dose-dense epirubicin, paclitaxel and cyclophosphamide
BCT: Breast conserving therapy
BCSS: Breast cancer-specific survival
ALH: Atypical lobular hyperplasia
LCIS: lobular carcinoma in situ
MRI: Magnetic resonance imaging
NME: Nonmass enhancement
ALND: Axillary lymph node dissection
SLNB: Sentinel lymph node biopsy
SLN: Sentinel lymph node
TAD: Targeted lymph node dissection
SERM: selective estrogen receptor modulator
AI: Aromatase inhibitor
SERD: Selective estrogen receptor downregulators
OFS: Ovarian function suppression
WES: Whole genome sequencing
PD: Disease progression
EMT: Epithelial-mesenchymal transition
TME: Tumor microenvironment
E-Ca: E-Cadherin
ESR1: Estrogen Receptor 1
ERRγ: Estrogen-related receptor γ
TAM: Tamoxifen
PI3K: Phosphatidylinositol 3-kinase
p120: p120-catenin
kiaso: Zinc finger transcription factor Kaiso
AP1: Activator protein 1
PTEN: Phosphatase and Tensin Homolog deleted on chromosome 10
mTOR: Neoadjuvant endocrine therapy
MYC: Cellular Myelocytomatosis oncogene
RTKs: Receptor tyrosine kinase
SCLC: Small cell lung cancer
HER3: Human epidermal growth factor receptor 3
FGFR4: Fibroblast growth factor receptor 4
IGF1R: Insulin-like growth factor 1 receptor
IGF1: Insulin-like growth factor 1
GRM/mGluR: Metabotropic glutamate receptors
MAPK: Mitogen-activated protein kinase
MEK: Mitogen-activated protein kinase kinase
FOXA1: Forkhead box protein A1
SNAIL: Zinc finger protein SNAIL
DSB: DNA double strand break
HDR: Homology-directedrepair
FA: Fanconi anemia
BER: Base excision repair
cAMP: Cyclic adenosine monophosphate
PKA: Protein kinase A
CREB: cAMP response element-binding protein
AKR1C1/2/3: Aldo-keto reductase family 1 member C1/C2/C3
SREBP1: Sterol regulatory element-binding protein 1
CAFs: Cancer-associated fibroblasts
LOXL1: Lysyl oxidase-like 1
Mrip: Myosin Phosphatase-Rho Interacting Protein
Rho: Ras homolog family member;
Rock: Rho-associated coiled-coil-containing protein kinase
Wnt11: Wnt family member 11
Akt: Protein kinase B
ADC: Antibody-drug conjugate
Ras: Ras GTPase
RAF: Rapidly Accelerated Fibrosarcoma kinase
ERK: Extracellular signal-regulated kinase
c- JUN: Jun proto-oncogene
c-FOS: Fos proto-oncogene
ELK: Ets-like gene transcription factor
ETS: E26 transformation-specific transcription factor
Cyclin D1: G1/S-specific cyclin-D1
E2: Estradiol
PARPi: Poly(ADP-ribose) polymerase inhibitor
SRE: Sterol regulatory element
RXR: Retinoid X receptor
CBP: CREB-binding protein
ATP: Adenosine triphosphate
BAPN: β-Aminopropionitrile (LOX inhibitor)
ECM: Extracellular matrix
RFS: Recurrence-Free Survival
OS: Overall Survival
CAP: Capecitabine
PFS: Progression-free survival
TAX: Taxanes
HIPEC: Hyperthermic intraperitoneal chemotherapy
RT: Radiotherapy
GI: Gestrointestine
Met: Metastasis
ROS1: ROS Proto-Oncogene 1
TILs: Tumor-infiltrating lymphocytes
PDL-1: Programmed Death-Ligand 1
ICIs: Immune Checkpoint Inhibitor
